# TRIM59 (Tripartite Motif-Containing 59) Expression in Pan-Cancer and Its Diagnostic and Prognostic Implications in Breast Cancer, Esophageal Cancer, Lung Squamous Cell Carcinoma, and Stomach Adenocarcinoma

**DOI:** 10.7759/cureus.101752

**Published:** 2026-01-17

**Authors:** Gamila A Attaelmanan, Mohamed Y Fateh Alrhman, Dina N Abdelrahman, Tarteel Mohamed, Mohanad Elkhidir, Roaa R Mohamed, Howaida Hamad, Marwa Mohamed, Elaf Siddig, Mawada Muzzammil, Mohamed Alfaki

**Affiliations:** 1 Medical Laboratory Sciences/Hematology, Al Neelain University, Khartoum, SDN; 2 Faculty of Medicine, National Ribat University, Khartoum, SDN; 3 Microbiology, Central Laboratory, Khartoum, SDN; 4 Community Medicine, Hamad Medical Corporation, Doha, QAT; 5 Faculty of Medicine, National University Biomedical Research Institute, Khartoum, SDN; 6 Faculty of Medicine, Ahfad University for Women, Khartoum, SDN; 7 Molecular Biology, Al Neelain University, Khartoum, SDN; 8 Faculty of Pharmacy, University of Khartoum, Khartoum, SDN; 9 Faculty of Pharmacy, University of Gezira, Khartoum, SDN; 10 Faculty of Medical Laboratory Sciences, University of Khartoum, Khartoum, SDN; 11 Computer Science, Al Neelain University, Khartoum, SDN

**Keywords:** bioinformatics, breast cancer (brca), esophageal cancer (esca), immune infiltration, lung squamous cell carcinoma (lusc), pan-cancer, stomach adenocarcinoma (stad), trim59

## Abstract

Background

Cancers remain a significant global health challenge; identification of cutting-edge diagnostic and prognostic markers plus innovative therapeutic targets is crucial. Tripartite motif molecule 59 (TRIM59) is a potential oncogene. However, its specific role in cancers is lacking. We aimed to investigate the expression of TRIM59 in pan-cancer as well as its implications for tumorigenesis, clinicopathological factors, and immune cell infiltration. Moreover, we aimed to investigate the protein-protein interaction and evaluate its potential as a diagnostic and prognostic biomarker in pan-cancer using bioinformatic analysis.

Methods

We analyzed TRIM59 across different cancer types using public datasets and bioinformatics tools. To study gene expression patterns, we used gene expression profiling interactive analysis (GEPIA), University of Alabama at Birmingham Cancer Data Analysis Portal (UALCAN), tumor immune estimation resource (TIMER), and University of California, Santa Cruz (UCSC) Xena, and validated our findings using additional datasets from the Gene Expression Omnibus (GEO) datasets. We assessed diagnostic performance with receiver operating characteristic (ROC) curve analysis. We also used Kaplan-Meier plotter (K-M plotter), GEPIA, UALCAN, and TIMER to explore how TRIM59 expression relates to patient prognosis and immune cell infiltration. Genetic changes were examined with cBioPortal, while the search tool for recurring instances of neighboring genes (STRING) and Kyoto Encyclopedia of Genes and Genomes (KEGG) analysis helped us study protein-protein interaction networks and pathway enrichment. For statistical analysis, we used the Limma (Linear Models for Microarray Analysis) program and standard tests.

Results

TRIM59 was significantly upregulated in 22 cancers, with the highest level of expression in breast cancer (BRCA), esophageal cancer (ESCA), lung squamous cell carcinoma (LUSC), and stomach adenocarcinoma (STAD). High TRIM59 expression is significantly associated with a worse prognosis in kidney renal papillary cell carcinoma (KIRP), liver hepatocellular carcinoma (LIHC), and lung adenocarcinoma (LUAD), and it was also associated with various clinicopathological factors in BRCA, ESCA, LUSC, STAD, and immune markers across all these cancers.

High TRIM59 expression is associated with infiltration of different immune cells in BRCA, ESCA, LUSC, STAD, LIHC, KIRP, and LUAD. Also, TRIM59 is associated with various genetic alterations across different types of tumors, and it interacts with several proteins.

Conclusion

In this study, we found that TRIM59 contributes to the carcinogenesis and increased malignant behavior of BRCA, ESCA, STAD, LUSC, LUAD, and KIRP. We also found that TRIM59 is a potential diagnostic biomarker for BRCA, ESCA, LUSC and STAD and a prognostic biomarker for KIRP, LIHC, and LUAD. Moreover, it correlates with immune infiltration and may be relevant to immunotherapy in these cancers. Furthermore, its correlation with tumor protein 53 (TP53), alpha/beta hydrolase domain containing 5 (ABHD5), and evolutionarily conserved signaling intermediate in toll pathway (ECSIT) may be used as a potential area of therapeutic intervention.

## Introduction

Cancer ranks second in the leading causes of death worldwide and is responsible for 9.7 million deaths in 2022 ​[1]​. Each minute, 11.8 people around the world die from cancer ​[2]​. Lung cancer emerged as the most prevalent cancer globally, followed by female breast cancer (BRCA), colorectal cancer, prostate cancer, and stomach cancer ​[1]​. Many genes have been linked with the oncogenesis of different cancer types, one of them is the tripartite motif (TRIM) protein family ​[3]​. More than 77 different proteins belong to the TRIM protein family, which is identified by the presence of an N-terminal region consisting of a RING domain, one or more B-box motifs, and a coiled-coil region ​[4]​. A novel TRIM family member called tripartite motif-containing 59 (TRIM59) ​[4]​ is connected to several malignancies, and higher TRIM59 expression is associated with increased cancer progression in pancreatic cancer ​[5]​. In non-small cell lung cancer, studies showed that TRIM59 is implicated in cell proliferation and metastasis, and TRIM59 knockdown resulted in cell cycle arrest in the G2 phase ​[6]​. Also, it was shown that prostate cancer tissues exhibited elevated levels of TRIM59, and TRIM59 knockdown dramatically reduced colony formation and cell cycle arrest at the S phase ​[7]​. Furthermore, patient TRIM59 scores are also associated with poor prognosis in cancer patients ​[8]​. Moreover, high TRIM59 expression was linked to 37 cancer types, including liver, skin, and endometrial cancers ​[4]​. TRIM59 has also been linked to esophageal, gastric, and breast malignancies ​[9-11]​. However, there is a lack of comprehensive analysis to establish the correlation between TRIM59 expression and tumorigenesis, diagnosis, and prognosis in these cancers. The current study used bioinformatics analysis to explore the TRIM59 expression in pan-cancer and investigate the relationship between the expression of TRIM59 and cancer tumorigenesis. Additionally, it evaluates the potential use of TRIM59 as a biomarker for diagnosis and prognosis in pan-cancer. The study also examines the association between TRIM59 expression and clinicopathological factors as well as immune cell infiltration in different types of cancers.

This article was previously presented as a meeting abstract at the ninth annual flagship research conference, Precision Medicine and Functional Genomics (PMFG), on November 11, 2023.

## Materials and methods

Gene expression analysis

GEPIA Database

Gene expression profiling interactive analysis (GEPIA; http://gepia.cancer-pku.cn/index.html) is a public database that is an interactive web resource based on GTEx and the Cancer Genome Atlas (TCGA) databases ​[12]. We used GEPIA to investigate the expression level of TRIM59 across pan-cancer and paired normal tissues. Furthermore, tumors with the highest TRIM59 expression were evaluated using an “expression box plot” module, where we matched TCGA normal and GTEx data. The cut-off of |Log2FC| (fold change) and p-value were 1 and 0.05, respectively.

UALCAN Database

The University of Alabama at Birmingham Cancer Data Analysis Portal (UALCAN; https://ualcan.path.uab.edu/) ​[13,14] is a web portal that provides analysis for OMICS data. The “pan-cancer view” module was used to explore the expression of TRIM59 across different types of tumors against normal TCGA samples. Moreover, the relationship between TRIM59 expression and clinical-pathological parameters (patient’s age, gender, race, cancer stages, cancer grades, TP53 mutation, *Helicobacter ​​​​​​pylori* infection, and menopause status) was conducted using the “expression” module in UALCAN.

TIMER Database

The expression level of TRIM59 was analyzed across different cancers and corresponding normal tissue through the use of the tumor immune estimation resource (TIMER), version 2.0 database (http://timer.comp-genomics.org) ​[15-17]​, which is a web resource for both comprehensive analysis and the clinical relevance of tumor-immune infiltrate identification.

UCSC Xena browser

We utilized the UCSC Xena browser (https://xenabrowser.net/) to access TCGA and GTEx data to validate the results obtained from GEPIA, UCLAN, and TIMER regarding the high expression of TRIM59 in breast cancer (BRCA), esophageal cancer (ESCA), lung squamous cell carcinoma (LUSC), and stomach adenocarcinoma (STAD). RNA-Seq gene expression data related to TRIM59 was selected, and relevant cancer cohorts were analyzed across these cancers. A box plot was created to illustrate the expression level of tumors versus normal tissue.

Receiver operating characteristic (ROC) curves

We utilize https://www.bioinformatics.com.cn/en to generate ROC curves to evaluate the performance of TRIM59 as a diagnostic marker in BRCA, ESCA, LUSC, and STAD. The area under the ROC curve (AUC) was calculated. 

Relationship between TRIM59 expression and prognosis in patients with cancer 

The correlation between TRIM59 expression and patient survival in pan-cancer compared to normal tissue was determined using the Kaplan-Meier plotter (K-M plotter; https://kmplot.com/analysis/) ​[11,18], GEPIA, and UALCAN.

Immune cell infiltration analysis

To investigate the potential role of TRIM59 in immune infiltration, we utilized the TIMER (https://cistrome.shinyapps.io/timer/) software. The “Gene module” and “survival module” were used to evaluate and visualize the levels of immune cell infiltration (purity, B cells, CD4+ T cells, CD8+ T cells, macrophages, neutrophils, and dendritic cells) and their correlation to overall survival in BRCA, ESCA, LUSC, STAD, KIRP, LIHC, and LUAD. The correlation between TRIM59 expression and immune cell infiltration was analyzed using Spearman's correlation coefficient.

Protein-protein interaction (PPI) gene enrichment analysis

PPI network analysis was conducted using the search tool for recurring instances of neighboring genes (STRING; https://string-db.org) to find the correlation and interaction between TRIM59 and its neighboring genes. The correlation between TRIM59 and these genes in BRCA, ESCA, LUSC, STAD, LUAD, LIHC, and KIRP was explored using the correlation module in the GEPIA database. Kyoto Encyclopedia of Genes and Genomes (KEGG) pathway enrichment analyses for the interacting genes were performed ​[19]​.

Genetic alteration

cBioPortal for Cancer Genomics (https://www.cbioportal.org/) was used to explore the genetic alteration of TRIM59 among pan-cancer using TCGA data; and K-M survival plot was also used to compare survival status between the genetically altered group and unaltered group.

TRIM59 expression validation

The Gene Expression Omnibus (GEO) database (https://www.ncbi.nlm.nih.gov/geo) was used to validate the results of our study [20]. We used six datasets from the GEO database, including two BRCA datasets (GSE29431 and GSE61304), one ESCA (GSE55857), two STAD (GSE118916 and GSE19826), and one LUSC dataset (GSE229509). Statistical analysis was carried out based on the criteria of |Log2FC|>0 and adjusted p-value < 0.05 to identify the differentially expressed genes. In addition, for obvious TRIM59 gene presentation, we used (https://www.bioinformatics.com.cn) to show the volcano plot that was related to those mentioned datasets.

Statistical data analysis

Various tests were used in statistical analyses, depending on the experimental design and data type. P-values for nonparametric and unequal variance data were determined using the Wilcoxon test and Welch's t-test, respectively. Data with equal variances and a normal distribution were subjected to the student's t-test. Mantel-Cox and log-rank tests were used for survival analysis. To evaluate linear relationships between variables, Pearson's correlation was employed. Fisher's exact test was used to analyze categorical data. The Limma (Linear Models for Microarray Analysis) tool was used to examine the microarray and gene expression data.

## Results

Pan-cancer analysis of TRIM59 expression across multiple databases

**Figure 1:**
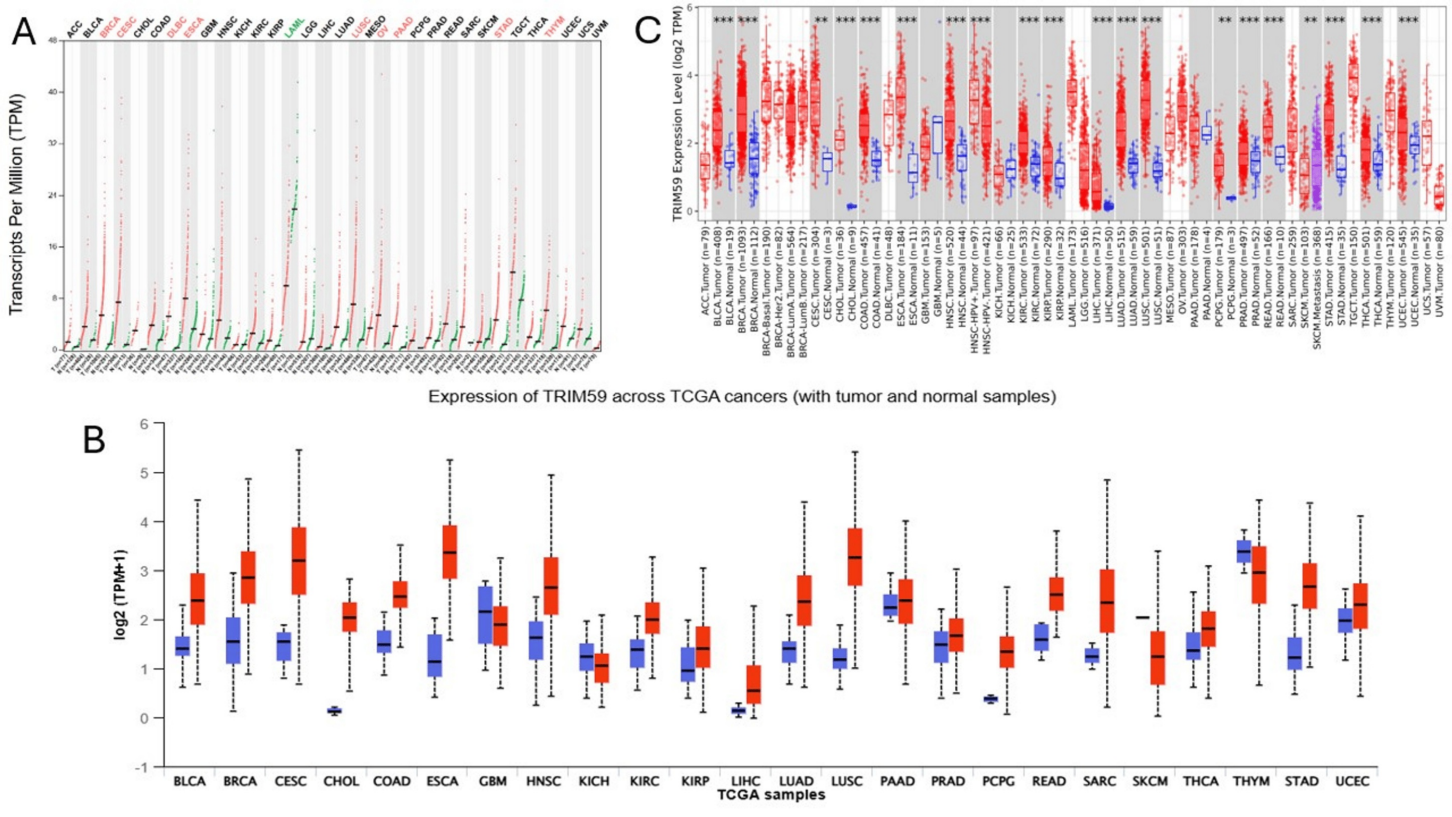
Graphical representation shows the difference of TRIM59 gene expression across different tumor tissue types (red) and normal tissue (blue) using multiple databases: (A) GEPIA, (B) UALCAN, and (C) TIMER P-value significance codes: ***0 ≤ P < 0.001, ** 0.001 ≤ P < 0.01. TRIM59: Tripartite motif molecule 59; GEPIA: Gene expression profiling interactive analysis; UALCAN: University of Alabama at Birmingham Cancer Data Analysis Portal; TIMER: Tumor immune estimation resource; TCGA: The Cancer Genome Atlas; BLCA: Bladder carcinoma; BRCA: Breast cancer; CESC: Cervical squamous cell carcinoma; CHOL: Cholangiocarcinoma; COAD: Colon adenocarcinoma; ESCA: Esophageal cancer; GBM: Glioblastoma multiforme; KICH: Kidney chromophobe carcinoma; HNSC: Head and neck squamous cell carcinoma; KIRC: Kidney renal clear cell carcinoma; KIRP: Kidney renal papillary cell carcinoma; LIHC: Liver hepatocellular carcinoma; LUAD: Lung adenocarcinoma; LUSC: Lung squamous cell carcinoma; PAAD: Pancreatic adenocarcinoma; PRAD: Prostate adenocarcinoma; PCPG: Pheochromocytoma and paraganglioma; READ: Rectal adenocarcinoma; SARC: Sarcoma; THYM: Thymoma; STAD: Stomach adenocarcinoma; SKCM: Skin cutaneous melanoma; THCA: Thyroid carcinoma; and UCEC: Uterine corpus endometrial carcinoma.

Investigation and analysis of the overall expression of the TRIM59 gene in pan-cancer compared to paired normal tissue were conducted utilizing GEPIA, UALCAN, and TIMER databases. The result revealed that TRIM59 is expressed in 33 different tumors; among these, the significant upregulation of TRIM59 is observed in nine tumors, including BRCA, cervical squamous cell carcinoma (CESC), DLBC, ESCA, LUSC, OV, pancreatic adenocarcinoma (PAAD), STAD, and thymoma (THYM), according to GEPIA (Figure 1, Panel A). On the other hand, the TRIM59 expression was significantly elevated in 20 cancers, including bladder carcinoma (BLCA), BRCA, CESC, cholangiocarcinoma (CHOL), colon adenocarcinoma (COAD), ESCA, head and neck squamous cell carcinoma (HNSC), kidney renal clear cell carcinoma (KIRC), KIRP, LIHC, LUAD, LUSC, PAAD, prostate adenocarcinoma (PRAD), pheochromocytoma and paraganglioma (PCPG), rectal adenocarcinoma (READ), sarcoma (SARC), STAD, thyroid carcinoma (THCA), and uterine corpus endometrial carcinoma (UCEC) based on UALCAN (Figure 1, Panel B). According to TIMER, the TRIM59 exhibited a significantly higher expression in 18 tumors, including BLCA, BRCA, CHOL, COAD, ESCA, HNSC, HNSC-HP+ve, KIRP, KIRC, LIHC, LUAD, LUSC, PRAD, READ, skin cutaneous melanoma (SKCM), STAD, THCA, and UCEC (Figure 1, Panel C).

**Figure 2:**
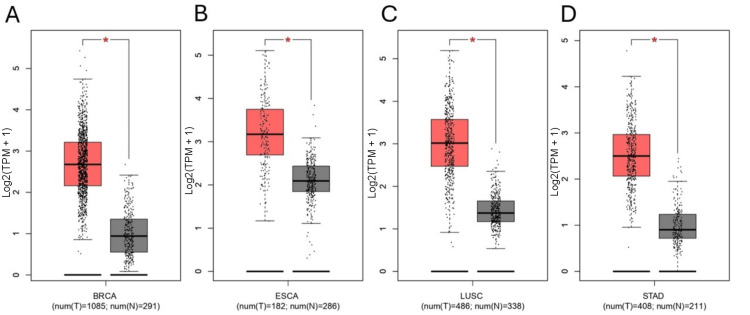
Boxplot showing TRIM59 expression in (A) breast cancer (BRCA), (B) esophageal cancer (ESCA), (C) lung squamous cell carcinoma (LUSC), and (D) stomach adenocarcinoma (STAD). Tumors (red) compared to normal tissue (gray) in the TCGA+GTEx dataset from GEPIA (*P-value < 0.05). TRIM59: Tripartite motif molecule 59; TCGA: The Cancer Genome Atlas; GEPIA: Gene expression profiling interactive analysis.

**Figure 3:**
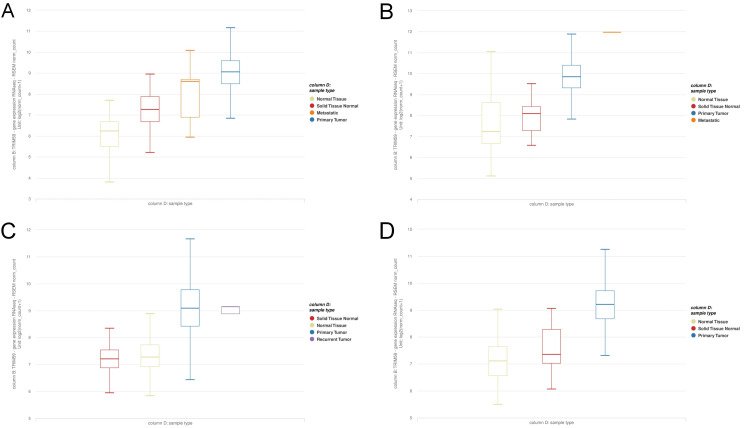
Xena browser showing TRIM59 expression in (A) breast cancer (BRCA), (B) esophageal cancer (ESCA), (C) lung cancer (LUSC), and (D) stomach adenocarcinoma (STAD). Primary tumor (blue) compared to normal tissue (yellow) and solid normal tissue (red) in TCGA TARGET GTEx samples. TRIM59: Tripartite motif molecule 59; TCGA: The Cancer Genome Atlas.

**Figure 4:**
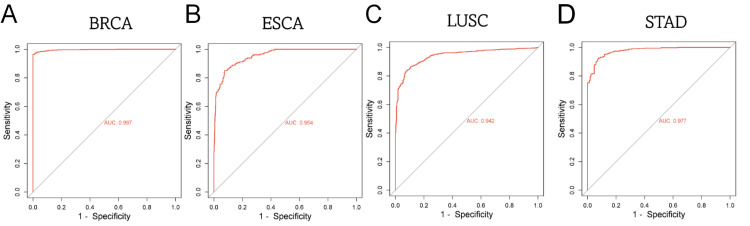
ROC curve showing the diagnostic efficacy of TRIM59 expression in (A) breast cancer (BRCA), (B) esophageal cancer (ESCA), (C) lung squamous cell carcinoma (LUSC), and (D) stomach adenocarcinoma (STAD) ROC: Receiver operating characteristic; TRIM59: Tripartite motif molecule 59.

Subsequently, to further confirm our findings, we proceeded to analyze the TRIM59 expression using GEPIA “boxplots” (Figure 2) and Xena browser “gene expression” (Figure 3) in tumors exhibiting the significant highest level of TRIM59 expression compared to their normal tissue, as verified by GEPIA, UALCAN, and TIMER (BRCA, ESCA, STAD, and LUSC). Eventually, receiver operator characteristic curve (ROC) was created to evaluate the diagnostic efficiency of TRIM59 in BRCA, ESCA, LUSC, and STAD, utilizing the pROC package, and the results showed that the area under the curve (AUC) of TRIM59 in BRCA, ESCA, LUSC, and STAD was 0.99, 0.0.95, 0.94, and 0.97, respectively, as presented in Figure 4. According to these findings, TRIM59 might contribute to BRCA, ESCA, LUSC, and STAD tumorigenesis and could be a potential diagnostic biomarker for these tumors.

The relationship between the TRIM59 expression and clinical characteristics of patients with BRCA, ESCA, LUSC, and STAD

**Figure 5:**
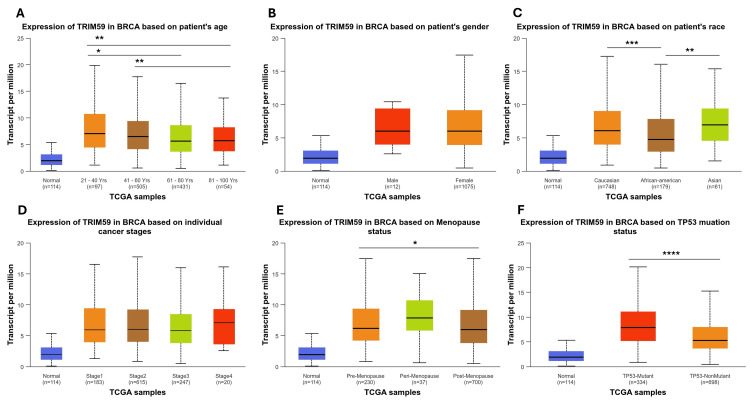
The relationship between the TRIM59 expression and clinical characteristics of BRCA patients from the TCGA database in UALCAN (A-F). *P < 0.05, **P < 0.01, ***P < 0.001. TRIM59: Tripartite motif molecule 59; BRCA: Breast cancer; TCGA: The Cancer Genome Atlas; UALCAN: University of Alabama at Birmingham Cancer Data Analysis Portal.

According to pan-cancer analysis results, BRCA, ESCA, LUSC, and STAD were selected for further analysis. We used UALCAN based on TCGA data to investigate the association between TRIM59 expression and clinical characteristics in these cancers. In BRCA, we explored the correlation between TRIM59 expression and age, gender, race, cancer stages, menopause status, and TP53 mutation status. We found a significant difference in TRIM59 expression levels across various age groups; patients aged between 21 and 40 years exhibited higher TRIM59 expression compared to patients aged 81-100 (P < 0.001) and 61-80 (P < 0.01). Similarly, patients in the age group between 41 and 60 years expressed higher TRIM59 when compared with those in the 81-100 age group (P < 0.001) (Figure 5, Panel A). For the criterion of race, the expression of TRIM59 was significantly higher in the Caucasian compared to African American (P < 0.001) and African American compared to Asian (P < 0.01) (Figure 5, Panel C). Furthermore, TRIM59 expression was higher in perimenopause patients compared to the post-menopause group (P < 0.01) (Figure 5, Panel E). Additionally, TRIM59 is significantly highly expressed in patients with TP53 mutation when compared to patients with no mutation (P < 0.0001) (Figure 5, Panel F), and there was no difference in TRIM59 expression between gender and across the various stage of BRCA (Figure 5, Panels B and D).

**Figure 6:**
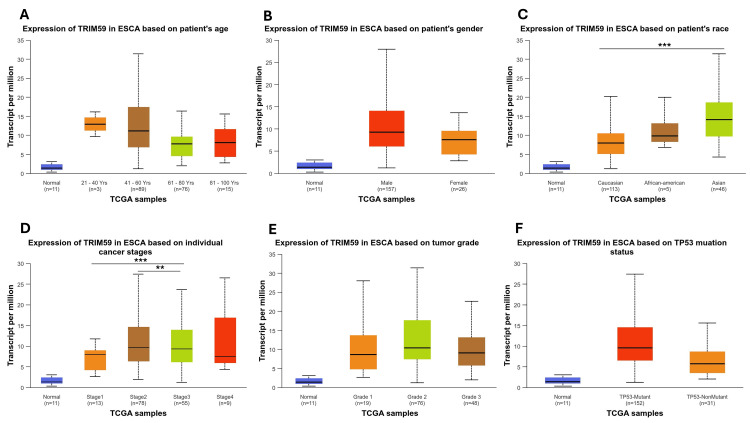
The relationship between the TRIM59 expression and clinical characteristics of ESCA patients from the TCGA database in UALCAN (A-F). *P < 0.05, **P < 0.01, ***P < 0.001. TRIM59: Tripartite motif molecule 59; ESCA: Esophageal cancer; TCGA: The Cancer Genome Atlas; UALCAN: University of Alabama at Birmingham Cancer Data Analysis Portal.

In ESCA, we found that TRIM59 expression was significantly higher in Caucasians compared to Asian patients with ESCA (P < 0.001) (Figure 6, Panel C). Regarding cancer stages, TRIM59 expression was considerably higher in patients with ESCA stage 3 than in patients in stage 1 (P < 0.001) and stage 2 (P < 0.01) groups (Figure 6, Panel D). On the other hand, there is no significant difference in TRIM59 expression between age group, gender, tumor grades, and TP53 mutation (Figure 6, Panels A, B, E, and F).

**Figure 7:**
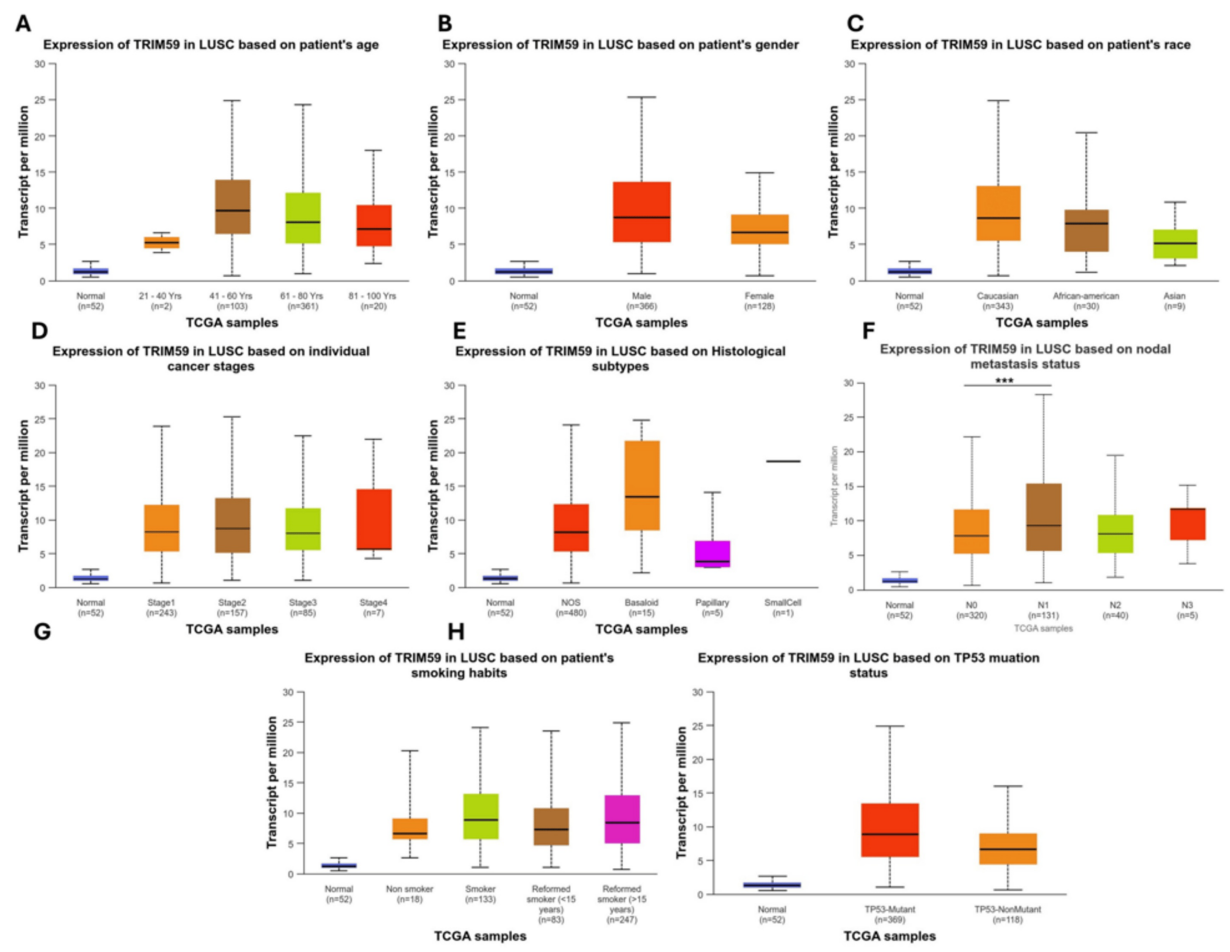
The relationship between the TRIM59 expression and clinical characteristics of LUSC patients from the TCGA database in UALCAN (A-H). *P < 0.05, **P < 0.01, ***P < 0.001. TRIM59: Tripartite motif molecule 59; LUSC: Lung squamous cell carcinoma; TCGA: The Cancer Genome Atlas; UALCAN: University of Alabama at Birmingham Cancer Data Analysis Portal.

In LUSC, the TRIM59 expression profile showed a significant difference based on nodal metastasis status. In patients with N1, there is a significantly high TRIM59 expression compared to patients with N0 (P < 0.001) (Figure 7, Panel F), while there is no significant difference in TRIM59 expression across other clinical factors, such as age, gender, race, cancer stages, histological subtypes, smoking status, and TP53 mutation status (Figure 7, Panels A-E, G, and H).

**Figure 8:**
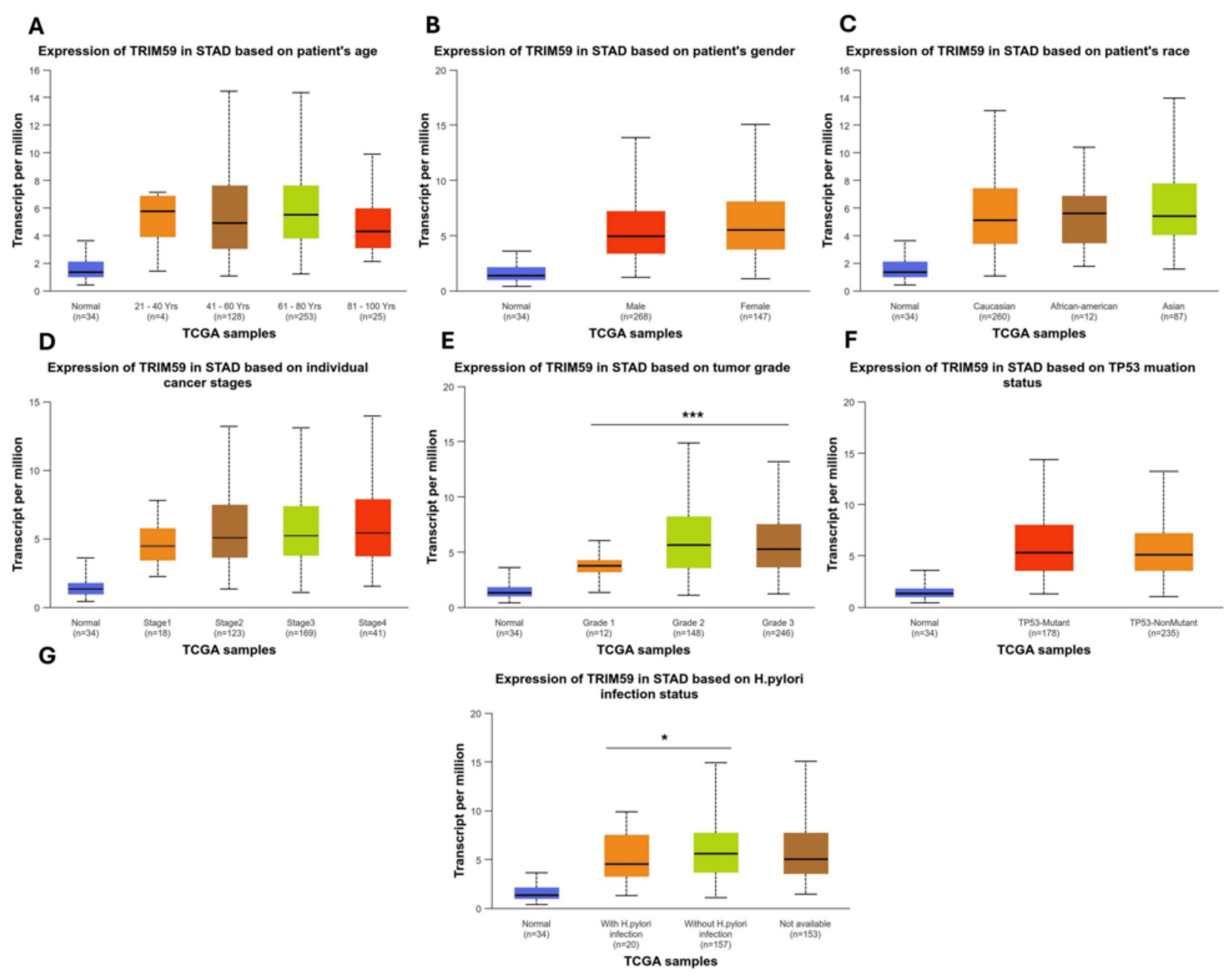
The relationship between the TRIM59 expression and clinical characteristics of STAD patients from the TCGA database in UALCAN (A-I). *P < 0.05, **P < 0.01, ***P < 0.00. TRIM59: Tripartite motif molecule 59; STAD: Stomach adenocarcinoma; TCGA: The Cancer Genome Atlas; UALCAN: University of Alabama at Birmingham Cancer Data Analysis Portal.

In STAD, we discovered that TRIM59 expression was substantially greater in patients with STAD grade 1 compared with those in grade 3 group (P < 0.001) (Figure 8, Panel E). TRIM59 expression was considerably higher in patients with *H. pylori* infection when compared to patients without *H. pylori* infection (P < 0.05) (Figure 8, Panel G). Regarding other clinicopathological factors (age, gender, race, stage, and TP53 mutation), no significant difference in TRIM59 expression was observed.

Relationship between TRIM59 expression and prognosis in patients with cancer

We used K-M plotter, GEPIA, and UALCAN databases to determine the correlation between TRIM59 expression and survival of patients across pan-cancer compared to normal tissues.

**Figure 9:**
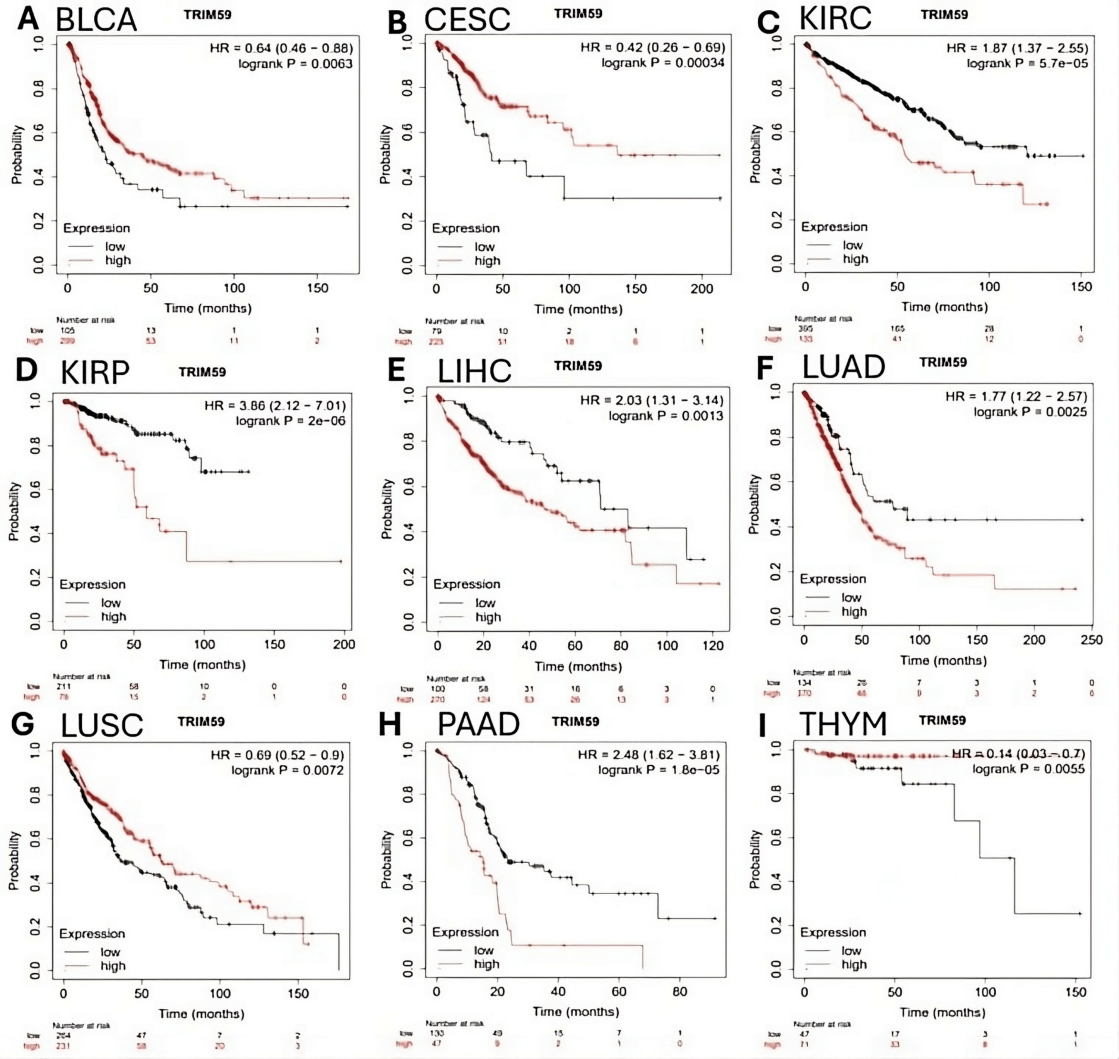
Kaplan-Meier (K-M) survival curves comparing the high (red) and low expression (black) of TRIM59 in different types of cancers in the K-M plotter databases. The hazard ratio (HR) estimation and long-rank test P-value are illustrated. Significant (log-rank test, P-value < 0.05). TRIM59: Tripartite motif molecule 59; BLCA: Bladder carcinoma; CESC: Cervical squamous cell carcinoma; KIRC: Kidney renal clear cell carcinoma; KIRP: Kidney renal papillary cell carcinoma; LIHC: Liver hepatocellular carcinoma; LUAD: Lung adenocarcinoma; LUSC: Lung squamous cell carcinoma; PAAD: Pancreatic adenocarcinoma; THYM: Thymoma.

In K-M plotter, a significant correlation was observed between TRIM59 expression and overall survival (OS) in patients with those nine cancers: BLCA (hazard ratio (HR) = 0.64, P = 0.0063), CESC (HR = 0.42, P = 0.00034), KIRC (HR = 1.87, P = 5.7E-05), KIRP (HR = 3.86, P = 2E-05), LIHC (HR = 2.03, P = 0.0013), LUAD (HR = 1.77, P = 0.0025), LUSC (HR = 0.69, P = 0.0072), PAAD (HR = 2.48, P = 1.8E-05), and THYM (HR = 0.14, P = 0.0055) (Figure 9). Additionally, we found that high TRIM59 expression was significantly associated with poor prognosis in patients with KIRC, KIRP, LIHC, LUAD, and PAAD. In contrast to patients with BLCA, CESC, LUSC, and THYM, the high expression of TRIM59 was associated with improved prognosis (Figure 9). On the other hand, no significant correlation was observed between TRIM59 expression levels and the prognosis of other types of cancer.

**Figure 10:**
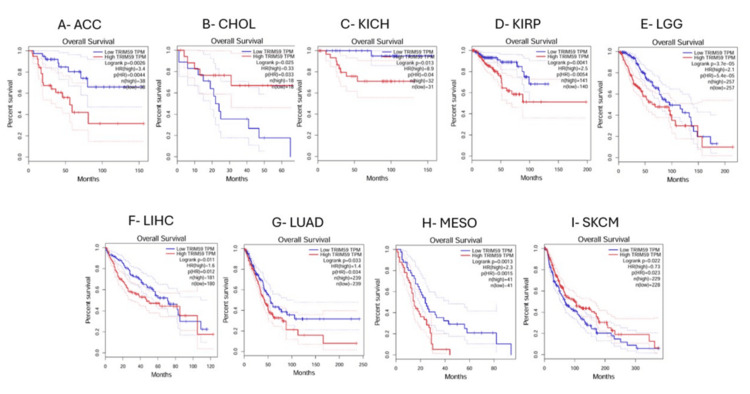
GEPIA survival analysis comparing the high (red) and low expression (blue) of TRIM59 in different types of cancers in the GEPIA databases. (A) ACC, (B) CHOL, (C) KICH, (D) KIRP, (E) LGG, (F) LIHC, (G) LUAD, (H) MESO, and (I) SKCM. The hazard ratio (HR) estimation and long-rank test p-value are illustrated. Significant (log-rank test, P-value < 0.05). GEPIA: Gene expression profiling interactive analysis; ACC: Adrenocortical carcinoma; CHOL: Cholangiocarcinoma; KICH: Kidney chromophobe carcinoma; KIRP: Kidney renal papillary cell carcinoma; LGG: Lower-grade glioma; LIHC: Liver hepatocellular carcinoma; LUAD: Lung adenocarcinoma; MESO: Mesothelioma; SKCM: Skin cutaneous melanoma.

In GEPIA, a significant correlation was observed between TRIM59 expression and OS in patients with adrenocortical carcinoma (ACC) (HR = 3.4, P = 0.0026), CHOL (HR = 0.33, P = 0.025), KICH (HR = 8.9, P = 0.013), KIRP (HR = 2.5, P = 0.0041), lower-grade glioma (LGG) (HR = 2.1, P = 3.7E-05), LIHC (HR = 1.6, P = 0.011), LUAD (HR = 1.4, P = 0.033), mesothelioma (MESO) (HR = 2.3, P = 0.0013), and SKCM (HR = 0.75, P = 0.022) (Figure 10). Additionally, we found that high TRIM59 expression was significantly associated with poor prognosis in patients with ACC, KICH, KIRP, LGG, LIHC, LUAD, and MESO. Conversely, high TRIM59 is significantly associated with good prognosis in patients with CHOL and SKCM (Figure 10). On the other hand, no significant correlation was observed between TRIM59 expression levels and the prognosis of other types of cancer.

**Figure 11:**
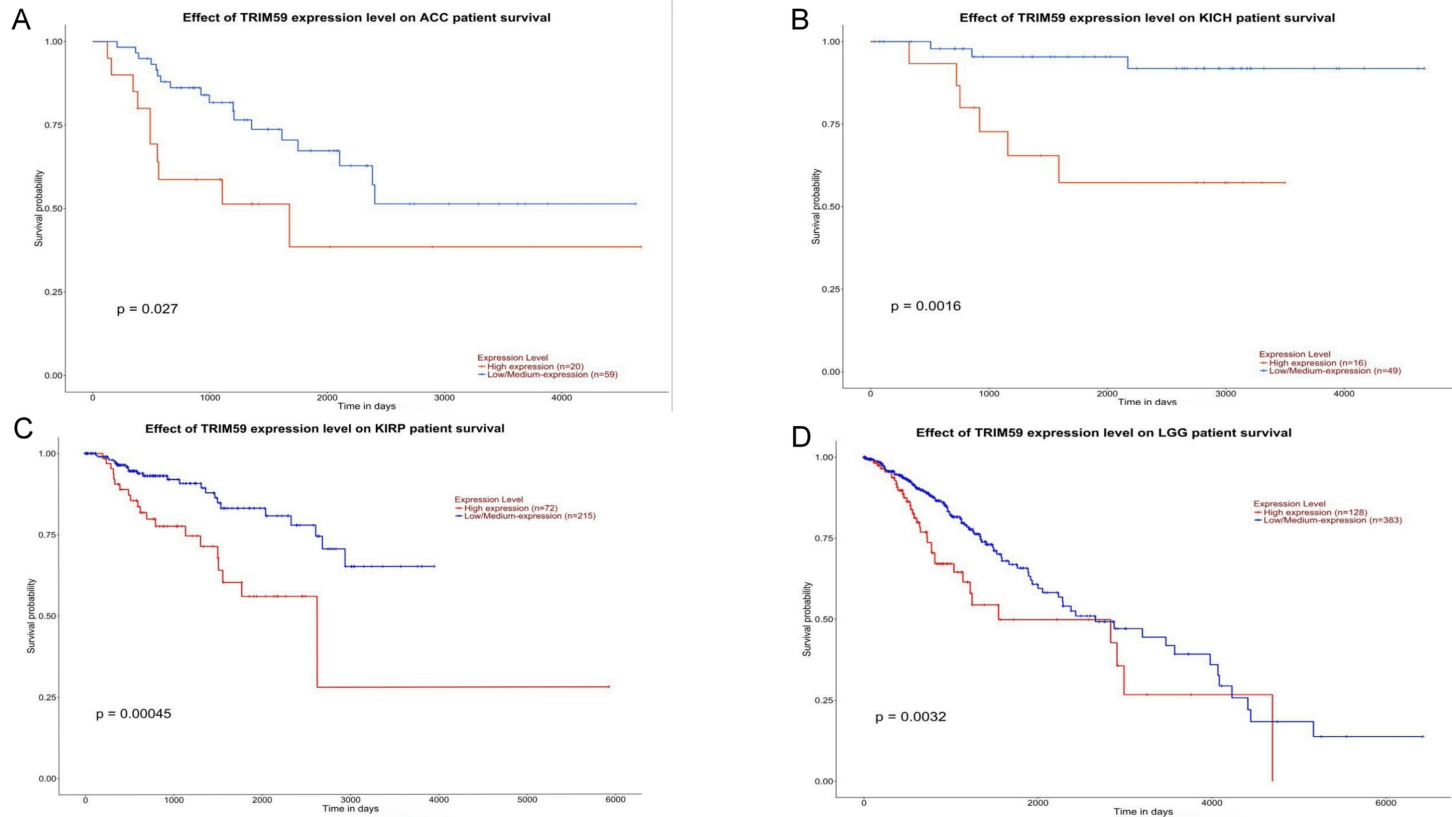
UALCAN survival analysis comparing the high (red) and low expression (blue) of TRIM59 in (A) ACC, (B) KICH, (C) KIRP, and (D) LGG in the UALCAN databases. Significant (P-value < 0.05). UALCAN: University of Alabama at Birmingham Cancer Data Analysis Portal; ACC: Adrenocortical carcinoma; KICH: Kidney chromophobe carcinoma; KIRP: Kidney renal papillary cell carcinoma; LGG: Lower-grade glioma.

**Figure 12:**
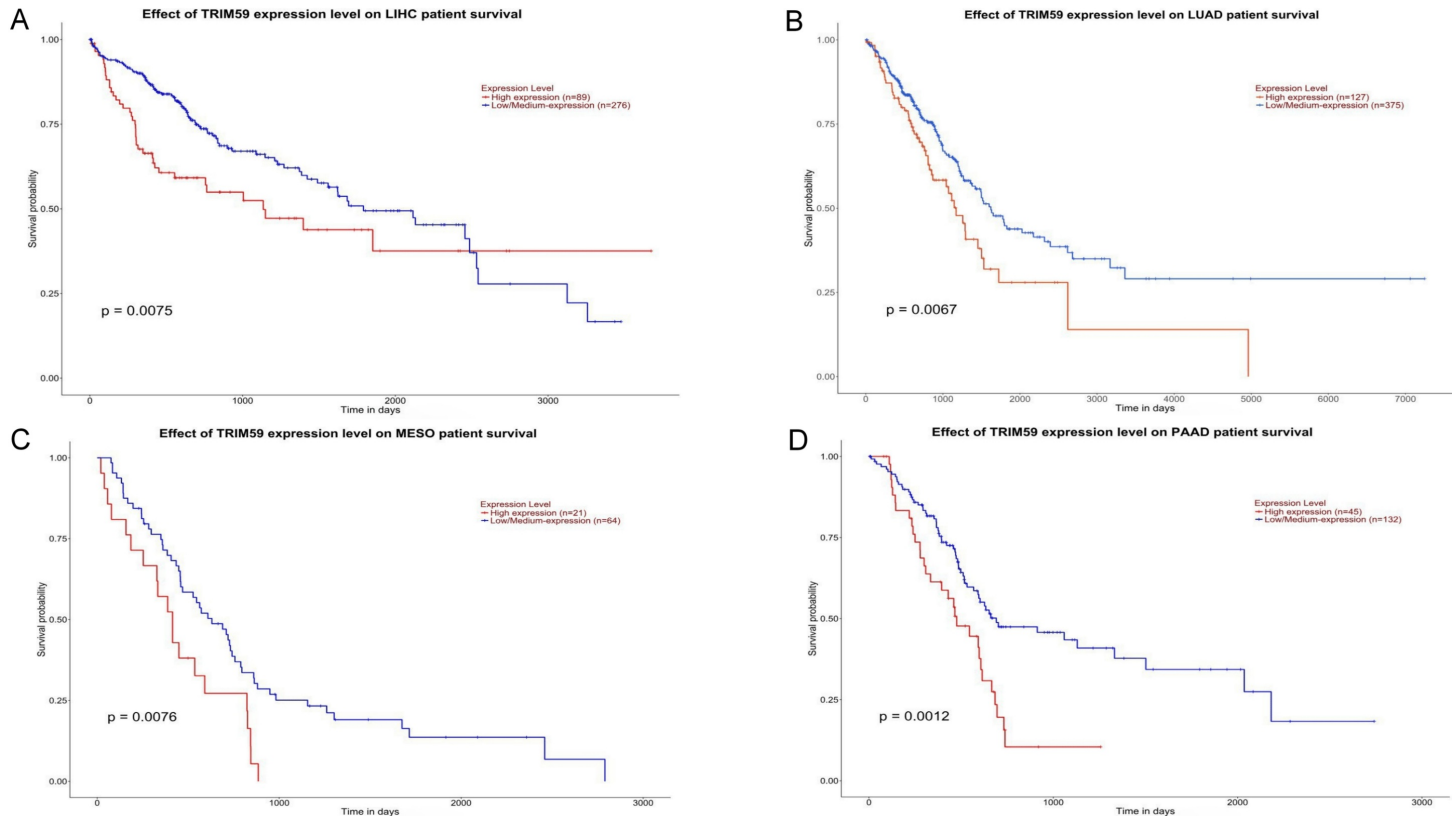
UALCAN survival analysis comparing the high (red) and low expression (blue) of TRIM59 in (A) LIHC, (B) LUAD, (C) MESO, and (D) PAAD in the UALCAN databases. Significant (P-value < 0.05). UALCAN: University of Alabama at Birmingham Cancer Data Analysis Portal; TRIM59: Tripartite motif molecule 59; LIHC: Liver hepatocellular carcinoma; LUAD: Lung adenocarcinoma; MESO: Mesothelioma; SKCM: Skin cutaneous melanoma; PAAD: Pancreatic adenocarcinoma.

**Figure 13:**
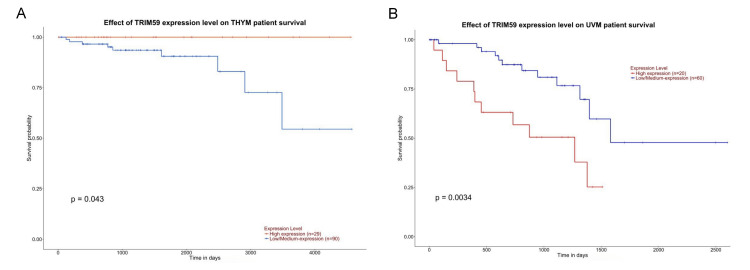
UALCAN survival analysis comparing the high (red) and low expression (blue) of TRIM59 in (A) THYM and (B) UVM in the UALCAN databases. Significant (P-value < 0.05). UALCAN: University of Alabama at Birmingham Cancer Data Analysis Portal; TRIM59: Tripartite motif molecule 59; THYM: Thymoma; UVM: Uveal melanoma.

In UALCAN, a significant correlation was observed between TRIM59 expression and OS in patients with LUAD (P = 0.0067), LGG (P = 0.0032), KIRP (P = 0.00045), LIHC (P = 0.0075), ACC (P = 0.027), KICH (P = 0.0016), UVM (P = 0.0034), THYM (P = 0.043), PAAD (P = 0.0012), and MESO (P = 0.0076) (Figures 11-13). Additionally, we observed that high TRIM59 expression was significantly associated with poor prognosis in patients with LUAD, LGG, KIRP, LIHC, ACC, KICH, UVM, PAAD, and MESO, whereas it was significantly associated with good prognosis in patients with THYM (Figures 11-13). On the other hand, no significant correlation was observed between TRIM59 expression levels and the prognosis of other types of cancer. Based on the cross-referenced analysis of K-M plotter, GEPIA, and UALCAN altogether, the results emphasized that high TRIM59 expression is significantly correlated with poor prognosis in KIRP, LIHC, and LUAD. This could reflect the potential of TRIM59 as a prognostic biomarker in these cancers.

Immune cell infiltration analysis

Association Between TRIM59 Expression and Immune Cell Infiltration in BRCA, ESCA, LUSC, and STAD

**Figure 14:**
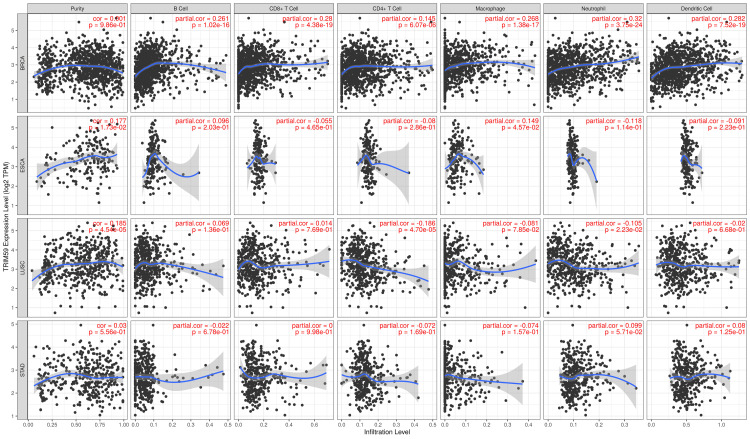
Correlation between TRIM59 expression and immune cell infiltration level in BRCA, ESCA, LUSC, and STAD. P-value < 0.05 is considered significant. TRIM59: Tripartite motif molecule 59; BRCA: Breast cancer; ESCA: Esophageal cancer; LUSC: Lung squamous cell carcinoma; STAD: Stomach adenocarcinoma.

We used the TIMER database to analyze the relationship between TRIM59 expression and the degree of immune cell infiltration in BRCA, ESCA, LUSC, and STAD (Figure 14) and found that in BRCA, high TRIM59 expression was significantly positively correlated with B cells (R = 0.26, P = 1.02E-16), CD8+ T cells (R = 0.28, P = 4.38E-19), CD4+ T cells (R = 0.14, P = 6.07E-06), macrophages (R = 0.26, P = 1.38E-17), neutrophils (R = 0.32, P = 3.75E-24), and dendritic cells (R = 0.28, P = 7.52E19). In ESCA, high TRIM59 expression was significantly positively correlated with purity (R = 0.17, P = 0.017) and macrophages (R = 0.14, P = 0.045). In LUSC, high TRIM59 expression was significantly positively correlated with purity (R = 0.18, P = 4.54E-05), while it was negatively correlated with CD4+ T cells (R = -0.18, P = 4.7E-05) and neutrophils (R = -0.1, P = 0.02). Regarding STAD, TRIM59 expression was significantly positively correlated only with neutrophil count (R = 0.09, P = 0.057).

**Figure 15:**
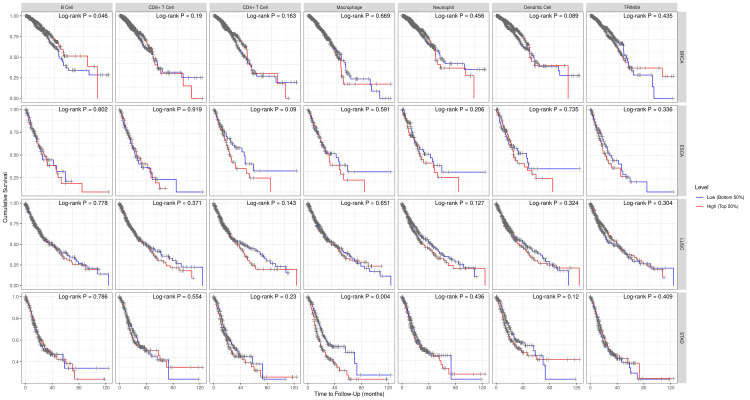
Kaplan-Meier survival analysis based on TRIM59 expression and immune cell infiltration level in BRCA, ESCA, LUSC, and STAD. P-value < 0.05 is considered significant. TRIM59: Tripartite motif molecule 59; BRCA: Breast cancer; ESCA: Esophageal cancer; LUSC: Lung squamous cell carcinoma; STAD: Stomach adenocarcinoma.

For survival analysis, we analyzed the correlation between TRIM59 expression and immune cell infiltration in patients with BRCA, ESCA, LUSC, and STAD by generating K-M plots using the TIMER database (Figure 15). The results demonstrated that B-cell infiltration was significantly correlated with a better prognosis of BRCA (P = 0.045), whereas a significant correlation was observed between macrophage infiltration and a worse prognosis of STAD (P = 0.004). However, in LUSC and ESCA, no significant correlation was noted.

Association Between TRIM59 Expression and Immune Cell Infiltration in KIRP, LIHC, and LUAD

**Figure 16:**
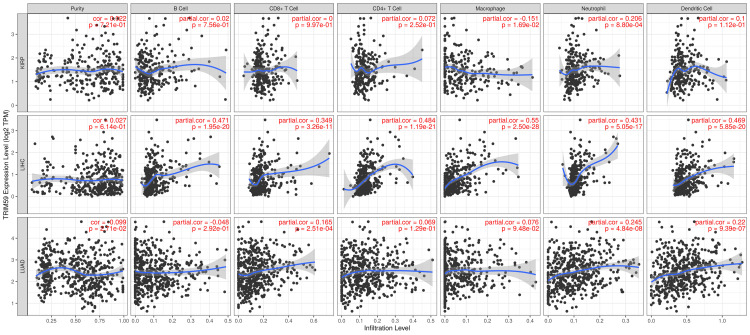
Correlation between immune cell infiltration and high TRIM59 expression in KIRP, LIHC, and LUAD TRIM59: Tripartite motif molecule 59; KIRP: Kidney renal papillary cell carcinoma; LIHC: Liver hepatocellular carcinoma; LUAD: Lung adenocarcinoma.

The correlation between high TRIM59 expression and immune cell infiltration in KIRP, LIHC, and LUAD was analyzed using the TIMER database to determine the immune cells involved. The result revealed that in KIRP, TRIM59 is significantly positively correlated with neutrophils (R = 0.2 P = 0.008). In LIHC, the TRIM59 is significantly positively correlated with all immune cells, B cells (R = 0.47, P = 1.95E-20), CD8+ T cells (R = 0.35, P = 3.2E-11), CD4+ T cells (R = 0.48, P = 1.19E-21), macrophages (R = 0.55, P = 2.5E-28), neutrophils (R = 0.43, P = 5.05E-17), and dendritic cells (R = 0.47, P = 5.8E-20). In LUAD, TRIM59 expression is significantly positively corelated with purity (R = 0.099, P = 0.02), CD8+ T cells (R = 0.6, P = 2.5E-4), neutrophils (R = 0.25, P = 4.84E-08), and dendritic cells (R = 0.22, P = 9.39E-07) (Figure 16).

**Figure 17:**
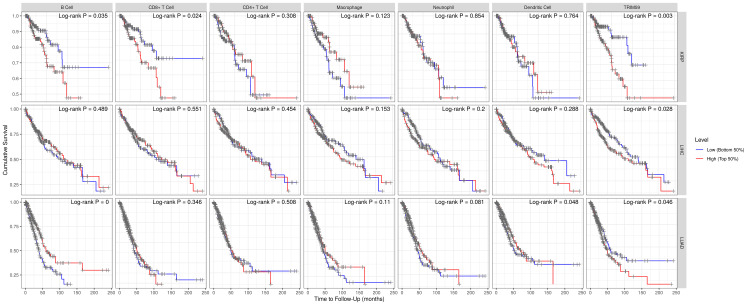
Survival analysis correlated with immune cell infiltration using the TIMER database for the three cancers that are significantly associated with overall survival in the three databases TIMER: Tumor immune estimation resource.

Regarding the survival analysis, in KIRP, B-cells and CD8+ T-cell infiltration were significantly correlated with poor prognosis (P = 0.035), (P = 0.024), respectively. In LIHC, there was no significant correlation between immune cell infiltration and prognosis. While in LUAD, dendritic cell infiltration was correlated with a better prognosis in the early stages of the disease followed by a poor prognosis in the late stages of the disease (P = 0.048). Moreover, B-cell infiltration was significantly correlated with a better prognosis (P = 0) (Figure 17).

PPI network and gene enrichment analysis of TRIM59 in BRCA, ESCA, STAD, LUSC, KIRP, LIHC, and LUAD

**Figure 18:**
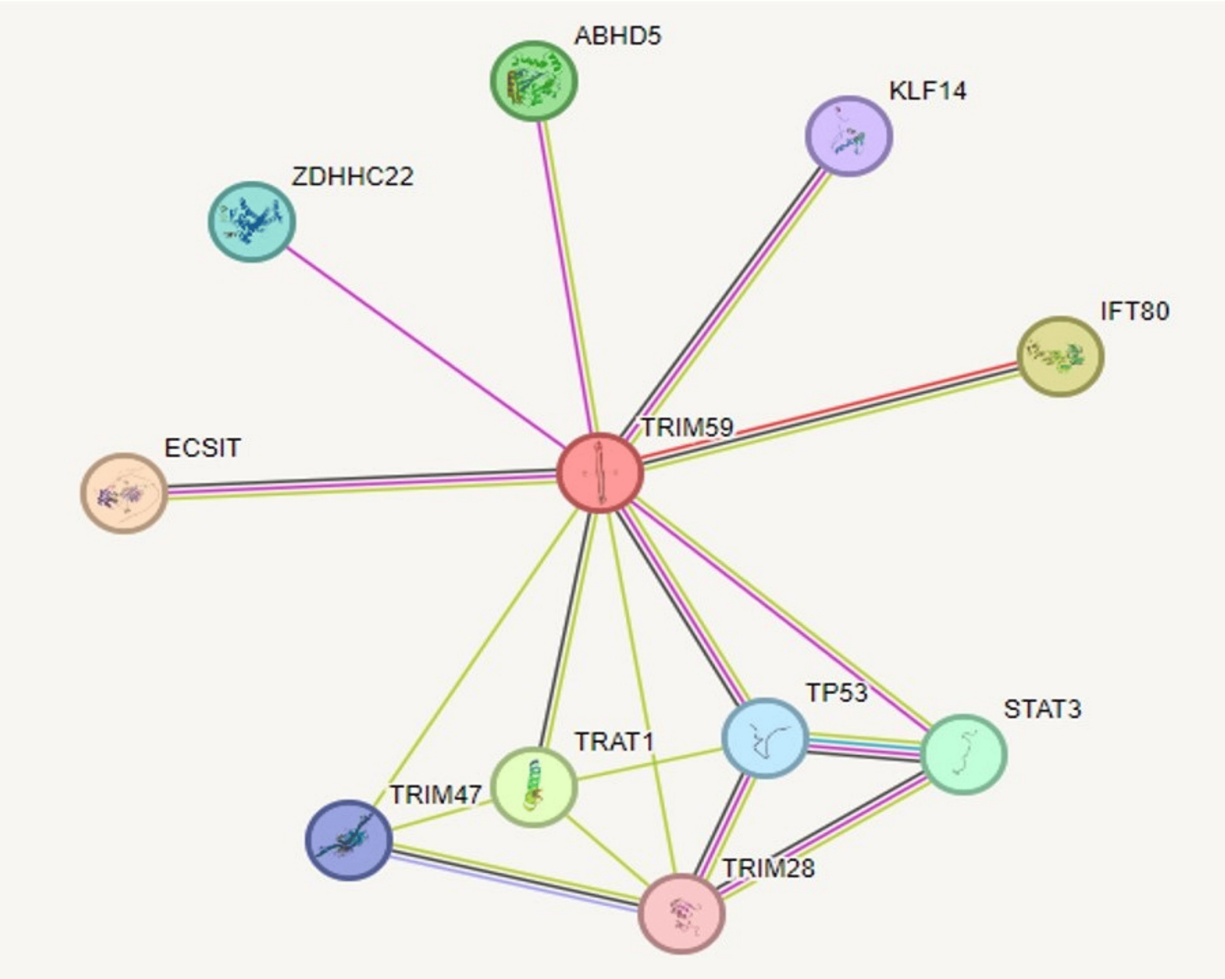
The PPI information about TRIM59 was evaluated by the STRING database showing the interaction between TRIM59 and the top 10 genes PPI: Protein-protein interaction; TRIM59: Tripartite motif molecule 59; STRING: Search tool for recurring instances of neighboring genes.

**Figure 19:**
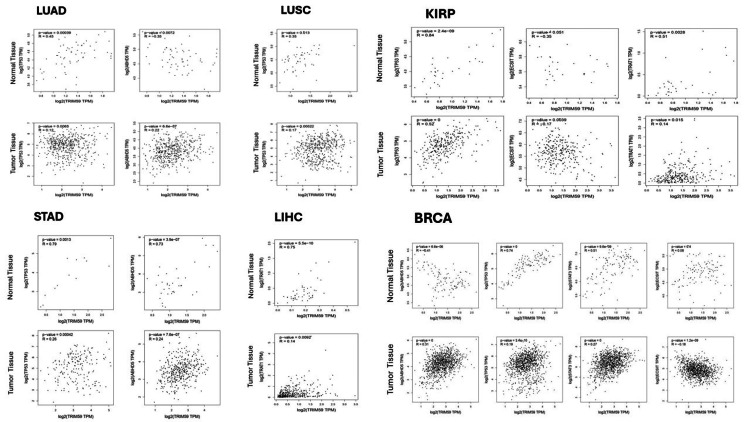
Expression correlation analysis of TRIM59 and interacting proteins in BRCA, STAD, LUAD, LUSC, LIHC, and KIRP using the GEPIA database. Pearson’s correlation was used, and a P-value of ≤ 0.05 was considered statistically significant. TRIM59: Tripartite motif molecule 59; GEPIA: Gene expression profiling interactive analysis; BRCA: Breast cancer; STAD: Stomach adenocarcinoma; LUAD: Lung adenocarcinoma; LUSC: Lung squamous cell carcinoma; LIHC: Liver hepatocellular carcinoma; KIRP: Kidney renal papillary cell carcinoma.

**Figure 20:**
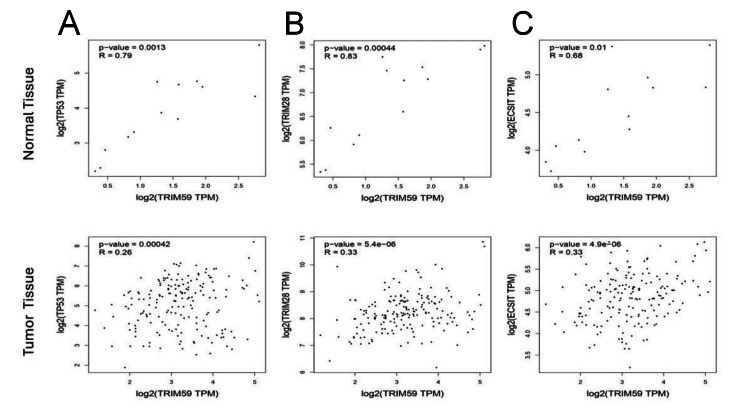
Expression correlation analysis of TRIM59, (A) TP53, (B) TRIM28, and (C) ECSIT in ESCA using the GEPIA database. Pearson’s correlation was used, and a P-value ≤ 0.05 was considered statistically significant. TRIM59: Tripartite motif molecule 59; ECSIT: Evolutionarily conserved signaling intermediate in toll pathway; ESCA: Esophageal cancer; GEPIA: Gene expression profiling interactive analysis.

STRING tool was employed to study the PPI information of TRIM59 and to obtain the top 10 TRIM59-interacting proteins. PPI web corresponded to 11 nodes and 17 edges. Each node pictured all proteins produced by an idiosyncratic protein-coding gene site, and each edge described the predicted functional associations. The interaction network of these 10 proteins is visualized in Figure 18. The correlations of TRIM59 with these genes (TP53, STAT3, TRIM28, TRIM47, ECSIT, ZDHHC22, ABHD5, KLF14, and IFT80) were investigated in BRCA, ESCA, LUSC, STAD, KIRP, LIHC, and LUAD using GEPIA (Figures 19, 20). The results showed that in BRCA, a significant correlation was observed between TRIM59 and TP53 (in normal tissue: R = 0.74, P < 0.001; in tumor tissue: R = 0.19, P = 3.4E-10), ABHD5 (in normal tissue: R = -0.41, P = 6.6E-06; in tumor tissue: R = 0.31, P < 0.001), STAT3 (in normal tissue: R = 0.51, P = 9.6E-09; in tumor tissue: R = 0.27; P < 0.001), and ECSIT (in normal tissue: R = 0.08, P = 0.4; in tumor tissue: R = -0.18; P < 1.2E-9). In STAD, a significant correlation was observed between TRIM59 and TP53 (in normal tissue: R = 0.63, P = 3.9E-05; in tumor tissue: R = 0.25, P = 2.1E-07) and ABHD5 (in normal tissue: R = 0.73, P = 3.9E-07; in tumor tissue: R = 0.24, P = 7.6E-07). In ESCA, a significant correlation between TRIM59 and TP53 (in normal tissue: R = 0.79, P = 0.0013; in tumor tissue: R = 0.26, P = 0.001), TRIM28 (in normal tissue: R = 0.83, P = 0.0004; in tumor tissue: R = 0.33, P = 5.4E-06), and ECSIT (in normal tissue: R = 0.68, P = 0.01; in tumor tissue: R = 0.33; P = 4.9E-06). In LUSC, a significant correlation was observed between TRIM59 and TP53 (in normal tissue: R = 0.35, P = 0.01; in tumor tissue: R = 0.17, P < 0.001). While in LUAD, TRIM59 was significantly correlated with ABHD5 (in normal tissue: R = -0.35, P = 0.0072; in tumor tissue: R = 0.22, P = 6.6E-07) and TP53 (in normal tissue: R = 0.45, P = 0.001; in tumor tissue: R = 0.12, P < 0.001). In KIRP, we noticed a significant correlation between TRIM59 and TP53 (in normal tissue: R = 0.84, P = 2E-04; in tumor tissue: R = 0.52, P = 0), ECSIT (in normal tissue: R = -0.35, P = 0.05; in tumor tissue: R = 0.17, P = 0.003), and TRAT1 (in normal tissue: R = 0.5, P = 0.002; in tumor tissue: R = 0.14, P = 0.015). In LIHC, there was a significant correlation between TRIM59 and TRAT1 only (in normal tissue: R = 0.75, P = 5.5E10; in tumor tissue: R = 0.14, P < 0.0092).

Regarding enrichment analysis, the KEGG database indicated significant enrichment of the TP53 pathway, which is one of the fundamental pathways of cancer development. Moreover, TRIM59 may be associated with breast and gastric cancer, and the nominee gene is TP53 in both cancers.

Genetic alteration analysis of TRIM59

**Figure 21:**
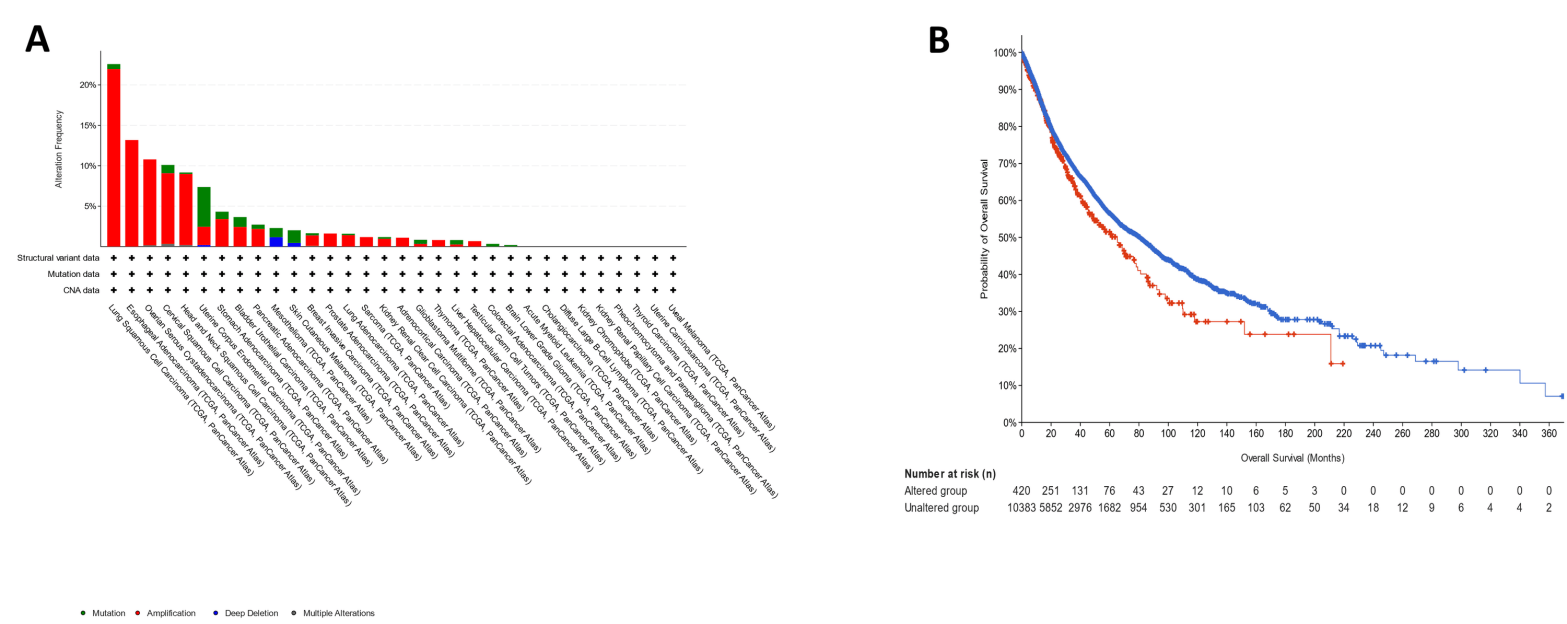
(A) Genetic alteration of TRIM59 across different types of cancers. (B) Survival analysis in patients with genetic alteration (red) and without genetic alteration (blue). TRIM59: Tripartite motif molecule 59.

We investigated the genetic alteration of TRIM59 in TCGA datasets (10967 samples/10953 patients) across different types of tumors utilizing the cBioPortal database. Several types of genetic changes in TRIM59 were observed including mutation, amplification, deep deletion, and multiple alteration as shown in Figure 21 (Panel A). Amplification is the most prevalent type of genetic alteration, mostly arising in LUSC, ESCA, ovarian cancer (OV), CESC, and HNSC. Among them, TRIM59 exhibited the highest amplification rate exceeding 23%, 13%, 11%, 10%, and 9% in patients with LUSC, ESCA, OV, CESC, and HNSC, respectively. On the other hand, mutations are the next most common genetic alteration, and they are mainly observed in uterine carcinoma (UCS), skin melanoma (SKCM), BLCA, mesothelioma, CESC, and STAD. Additionally, we demonstrated the relationship between genetic alteration in TRIM59 and the clinical feature of clinical survival prognosis through several types of cancers. As presented in Figure 21 (Panel B), we compared patients with genetic alteration with patients without genetic alteration, we noted that patients with genetic alteration have a worse prognosis in OS (P = 0.01). 

Validation analysis

**Figure 22:**
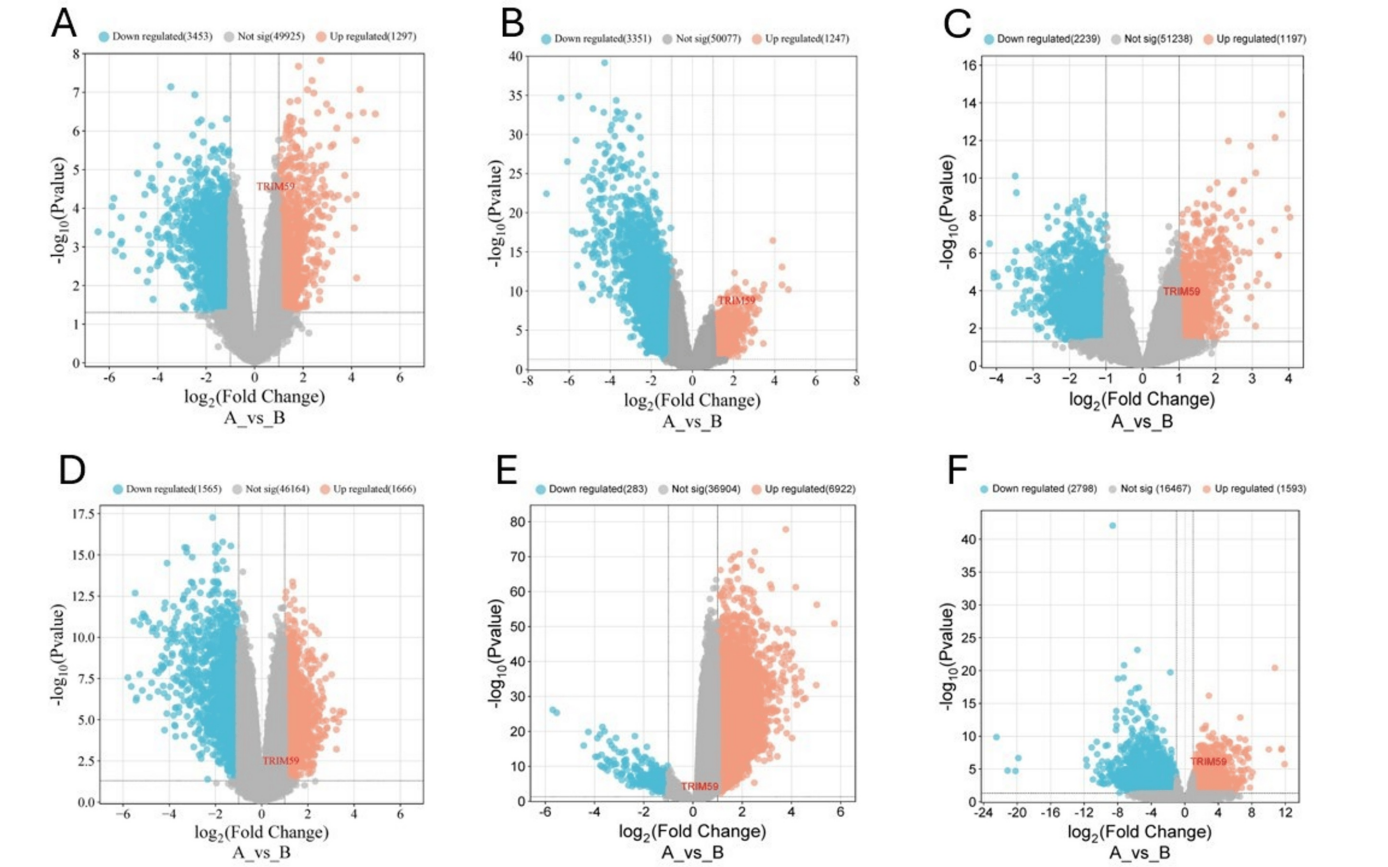
Volcano plot shows upregulated (orange) and downregulated genes (turquoise) in (A) breast cancer GSE61304 dataset, (B) breast cancer GSE29431 dataset, (C) stomach cancer GSE19826 dataset, (D) stomach cancer GSE118916 dataset, (E) esophageal cancer GSE55857 dataset, and (F) lung squamous cell carcinoma GSE229509 dataset with TRIM59 as an upregulated gene TRIM59: Tripartite motif molecule 59.

In order to validate and consolidate the reliability of our findings, further statistical analysis was performed on multiple datasets obtained from the GEO, and the results consistently exhibited a similar pattern that supports our results (Table 1 and Figure 22).

**Table 1:** The significant alteration in TRIM59 expression between cancerous and normal tissue in the GEO database. Limma’s moderated t-test was used to calculate the P-values. TRIM59: Tripartite motif molecule 59; GEO: Gene Expression Omnibus.

Types of cancer	Dataset	Normal sample	Tumor sample	TRIM59 regulation	P-value
Breast cancer (BRCA)	GSE29431	12	54	Up	2.05E-09
	GSE61304	4	58	Up	5.76E-03
Esophageal cancer (ESCA)	GSE55857	108	216	UP	0.0013
Stomach adenocarcinoma (STAD)	GSE118916	15	15	Up	0.01
	GSE19826	15	12	Up	0.008
Lung squamous cell carcinoma (LUSC)	GSE229509	3	23	Up	1.01E-6

## Discussion

Cancer has long been a leading cause of worry for the universe's health and places a significant financial burden on both individuals and the healthcare system. Our study found that TRIM59 is involved in several cancers, including BRCA, ESCA, STAD, and LUSC [9-11]. A set of genes with aberrant expression that may be exploited as diagnostic markers or prognostic predictors is always associated with cancers ​[21]​.

TRIM59 is known to affect normal cellular proliferation through stimulation of post-translational modifications of diverse substrates that affect a variety of cellular activities, like cell growth, development, differentiation, death, inflammation, and immunity. In addition, TRIM59 controls protein expression and also affects the tumor cells' aggressive behavior ​[22]​. Moreover, at the clinical experiments level, TRIM59 was discovered in mouse prostate cancer models as an early signal transducer in SV40 Tag and Ras oncogenic pathways ​[23]​.

Pan-cancer analysis of TRIM59 expression

This study analyzed the expression of TRIM59 in various human tumors using data from the TCGA and GTEx databases, and we found that TRIM59 expression is upregulated in 22 types of solid tumors when compared to adjacent normal tissues. This result is consistent with a previous study conducted by Jin et al. in 2021 ​[21] that demonstrated TRIM59 upregulation in 15 types of tumors. These findings suggested that TRIM59 may be pro-carcinogenic and could be a promising diagnostic biomarker for these tumors. Interestingly, we found that the highest levels of TRIM59 are displayed by BRCA, ESCA, LUSC, and STAD; therefore, we used a variety of public databases to close the knowledge gap and investigate the function of TRIM59 in these tumors.

The relationship between TRIM59 expression and clinicopathological factors in BRCA, ESCA, LUSC, and STAD

Our study investigated the relationship between TRIM59 expression and clinicopathological factors in BRCA, ESCA, LUSC, and STAD. In BRCA, we found a significant correlation between high TRIM59 expression and age, race, menopausal status, and TP53 mutation. We found that TRIM59 expression was significantly higher in BRCA patients with TP53 mutations than those without mutations. This finding is consistent with a study done by Liu et al., who reported a similar association in BRCA, suggesting a potential cooperation between TRIM59 and TP53 loss-of-function to enhance tumor progression [11]. Our results also demonstrated a significantly higher gene expression in younger BRCA patients than in elderly patients. This finding suggests a potential correlation between elevated expression of TRIM59 and early-onset BRCA in younger ages. As has been previously mentioned, TRIM59 overexpression was involved in oncogenic pathways associated with proliferation, invasion, and survival, which could explain the aggressive tumor biology that is often present in younger patients [24].

A previous study done by Horvath in 2013 reported that TRIM59 has been identified as one of the genes contributing to the epigenetic aging clock, which estimates age based on DNA methylation [25]. Accordingly, TRIM59 may serve as a cellular aging marker, and its higher expression observed in younger patients may reflect its role in early tumorigenesis rather than tumor progression related to aging. Furthermore, in the present study, we observed that TRIM59 expression was significantly higher in perimenopausal patients compared to the postmenopausal group. This finding suggests that TRIM59 may be affected by hormonal fluctuations associated with the menopausal transition. In the perimenopausal stage, there are varying levels of circulating estrogen and progesterone, whereas in post-menopause, a marked decline in these hormones occurs [26]. Considering that estrogen and progesterone are key regulators in breast tissue biology, it is likely that TRIM59 expression may be modulated by these hormones. Although there is a lack of previous studies that link the menopausal status to TRIM59 expression directly, our results are supported indirectly by a study done by Liu et al. who demonstrated that TRIM59 enhances BRCA cell progression via activation of the AKT pathway, which is known to crosstalk with estrogen signaling pathways [11]. These findings imply that TRIM59 may be more active in hormone-responsive tumors and could play a role in the aggressive tumor phenotype seen in perimenopausal patients. Further research is needed to define the precise mechanisms through which hormonal changes modulate TRIM59 and also to explore its potential as a prognostic marker or therapeutic target in hormone-sensitive breast cancers.

Moreover, we found that TRIM59 was highest in Caucasian patients compared to African Americans and in African Americans compared to Asians in BRCA, while in ESCA, TRIM59 was significantly higher in Caucasians compared to Asians. This variation could reflect genetic, epigenetic, or environmental factors influencing TRIM59 regulation, which may contribute to biological differences in tumor behavior across different races. Additionally, we noted that TRIM59 expression was significantly higher in stage 3 compared to stages 1 and 2 in ESCA, indicating its potential contribution to tumor progression. This is supported by Liu et al.'s finding, which reported that TRIM59 promotes ESCA progression and invasiveness [9]. Similarly, another study by Liu et al. showed that knockdown of TRIM59 in ESCA cells led to increased expression of p53, which in turn reduced cell viability, migration, and invasion, highlighting TRIM59 oncogenic role [27]. All together, these results support that TRIM59 could be a potential biomarker for advanced disease stages and a therapeutic target in ESCA; however, more research is needed to explore its underlying mechanisms. Moreover, in LUSC, our results showed that TRIM59 expression was significantly higher in patients with N1 nodal metastasis compared to N0. This result suggests that TRIM59 may contribute to tumor invasion to the lymph node and could serve as a biomarker for early metastatic progression in LUSC. This finding agrees with prior studies, where TRIM59 overexpression was associated with enhancement of metastasis, tumor cell proliferation, and poor prognosis in lung cancer [28].

Furthermore, in the analysis of TRIM59 expression in STAD, we observed that TRIM59 expression was significantly higher in grade 1 tumors compared to grade 3. This finding suggests that TRIM59 may play a role in the early stages of gastric tumor development, while previous studies have reported a positive correlation between TRIM59 expression and tumor grade in gastric cancer [10]; this may explain the complex mechanism by which TRIM59 contributes to tumor differentiation and progression. Additionally, we found that TRIM59 expression was significantly elevated in patients with Helicobacter pylori (H. pylori) infection compared to those without the infection. The increased TRIM59 expression in H. pylori-positive patients may reflect its involvement in infection-driven gastric tumorigenesis. Previous studies in this context are limited, so further research is needed to clarify the mechanisms by which TRIM59 is involved in H. pylori-associated gastric cancer development.

Relationship between TRIM59 expression and survival in patients with cancer

This study investigated the relationship between TRIM59 expression and prognosis in patients with cancer. We found that high TRIM59 expression is associated with poor prognosis in patients with KIRP, LIHC, and LUAD. This result is consistent with a previous study, which showed that high TRIM59 expression indicates poor prognosis in KIRP and LUAD ​[21]​. This could reflect the potential of TRIM59 as a prognostic biomarker in these cancers.

Association between TRIM59 expression and immune cell infiltration in BRCA, ESCA, LUSC, STAD, KIRP, LIHC, and LUAD

Components of tumor-infiltrating immune cells can be used as a biomarker for predicting response to treatment and patient survival in terms of chemotherapy and immunotherapy, thus cancer’s immune cell infiltration is directly correlated to clinical outcomes ​[29]​. This study analyzed the correlation between TRIM59 expression and immune cell infiltration in BRCA, ESCA, LUSC, and STAD, considering the importance of the tumor microenvironment and its crucial role in predicting clinical findings, diagnosis, and treatment of cancer. We found that in BRCA, high TRIM59 expression was highly related to CD4+ T-cells, macrophages, B-cells, CD8+ T-cells, dendritic cells, and neutrophils, and it is also significantly correlated with a better prognosis with B-cell infiltration (P = 0.045), which suggests a potentially favorable immune microenvironment associated with TRIM59 expression in BRCA. In ESCA, high TRIM59 expression was highly related to purity and macrophages, unlike STAD in which high TRIM59 expression was significantly related to neutrophils, and it expressed poor prognosis related to macrophage (P = 0.004). Zhou et al.'s findings are compliant with our study; they confirmed that TRIM59 aggravates the ubiquitination and degradation of P53 (a functional transcription factor in activated T cells), thus promoting gastric carcinogenesis ​[10]. While in LUSC, it is positively correlated with purity and negatively correlated with CD+4 T-cell and neutrophil. Our results are further supported by Liang et al., who reported that TRIM59 is expressed in exosomes derived from lung cancer cells and leads to lung cancer progression via macrophage activation ​[30]​.

Moreover, our study investigated the relationship between TRIM59 expression and immune cell infiltration in KIRP, LIHC, and LUAD. We found in KIRP that high TRIM59 expression was significantly corelated with neutrophil and associated with decreased survival related to B-cell and CD8+ T-cell infiltration. On the other hand, in LIHC, TRIM59 was correlated with all immune cell infiltration, but there was no significant correlation regarding the survival rate. In LUAD, high TRIM59 expression is significantly corelated with purity, CD8 T-cell, neutrophil, and dendritic cell, which is associated with better prognosis in the early months but a worse prognosis in later months. B-cell is associated with better survival outcome, and this could be used as a target for immunotherapy to increase survival rates. A study conducted by Yang et al. in 2021 showed that TRIM59 expression is significantly positively corelated with dendritic cells infiltration (P = 9.39 × 10^7^) in LUAD ​[31]. According to our knowledge, there is a lack of studies that evaluated the role of TRIM59 expression on immune cell infiltration in these cancers in particular, and our study is among the first to investigate this relationship in these cancers. However, more studies are needed to further understand these complex mechanisms, so it could be a possible chance for developing new therapeutic strategies.

PPI and gene enrichment analysis of TRIM59 in BRCA, ESCA, STAD, LUAD, LUSC, LIHC, and KIRP

In this study, PPI was carried out through the KEGG pathway utilizing the STRING database and found that ECSIT, KLF14, ABHD5, STAT3, TRIM47, TRIM28, TRAT1, ZDHHC22, IFT80, and TP53 were the functional genes with TRIM59. Using the GEPIA correlation test, we observed a variation in the direction and degree of the correlation between TRIM59 and these genes among normal and tumor tissue in BRCA, ESCA, STAD, LUAD, LUSC, KIRP, and LIHC.

In BRCA and LUAD, we found an increase in the correlation degree or shift to a positive between TRIM59 and ABHD5 in tumor tissue compared to normal tissue. On the other hand, in STAD, the correlation between TRIM59 and ABHD5 was found to be decreased. Previous studies have established a connection between ABHD5 and macrophages in tumor tissue. They reported that TRIM59 facilitates the progression of lung cancer through ubiquitination and subsequent degradation of ABHD5 leading to macrophage reprogramming and NLRP3 inflammasome pathway activation ​[30]. Our results suggest that a similar mechanism may occur in STAD, as we demonstrated a significant correlation between macrophage infiltration and poor prognosis in STAD patients with high TRIM59 expression. Conversely, previous research reported that ABHD5 has a tumor-suppressive role in colorectal and prostate cancer ​[32,33];​ this finding may support the positive correlation between TRIM59 and ABHD5 that we observed in BRCA and LUAD. However, it is crucial to conduct more research to investigate this correlation carefully in BRCA, LUAD, and STAD, as it may be a potential area for therapeutic intervention.

Moreover, we noticed a decrease in the correlation level between TRIM59 and TP53 in BRCA, ESCA, STAD, LUSC, LUAD, and KIRP among tumor tissue compared to normal tissue. Notably, this finding aligns with the result of KEGG pathway analysis, which demonstrated a significant enrichment of TP53 in BRCA and STAD. This finding is consistent with previous studies showing that the high expression of TRIM59 increased the ubiquitination and degradation of P53, particularly in breast and gastric cancer, which in turn leads to a poor prognosis and an increase in malignant behavior by controlling the AKT pathway ​[11,25].

Our study also identified that there is a decline in the correlation between TRIM59 and STAT3 in BRCA, and a previous study showed that STAT3 is aberrantly activated in several malignancies, including BRCA [34]. Other studies reported that TRIM59 induces activation of STAT3 and promotes carcinogenesis and drug resistance of multiple types of cancer through EGFR/STAT3 signaling pathway [23,35]. Taken together, our study indicates that TRIM59 overexpression might contribute to the carcinogenesis of BRCA via STAT3 regulation. It is worth pointing out that there is a contradiction in the role of TRIM59 in BRCA due to its conflicting action on the different genes and signaling pathways in BRCA, so more research and careful investigation are essential to reveal its specific role in BRCA and its potential as a therapeutic target.

Likewise, we detected a decrease in the correlation between TRIM59 and TRIM28 in ESCA tumor tissue compared to normal tissue. So far, no study has investigated the correlation between TRIM59 and TRIM28 in ESCA, but a previous study showed that TRIM28 and TRIM47 were over-expressed in BRCA together with TRIM59 [36].

Moreover, we noticed a decrease in the relationship between TRIM59 and ECSIT in BRCA, ESCA, and KIRP. Although this correlation in cancer remains unexplored, a study has revealed that ECSIT participates in RIG-I-like receptors (RLR) pathway [37], and TRIM59 has been recognized as a potential suppressor of RLR-induced activation of interferon regulatory factors (IRFs) and nuclear factor-κB (NF-KB) through interaction with ECSIT [38]. On the other hand, another study has reported that activation of RLRs can directly stimulate cancer cell death [39]. Collectively, our results suggest that the overexpression of TRIM59 might contribute to cancer progression by modulating ECSIT and the RLR pathway inhibition.

Additionally, we found that TRIM59 interacts with TRAT1 in both LIHC and KIRP. Previous studies have revealed that TRAT1 plays an essential role in T-cell receptor (TCR) signaling, which is crucial for T-cell activation against tumors [40]. Our results showed that these two genes are significantly and strongly correlated in normal tissue, suggesting that TRIM59 might be involved in immune suppression-related pathway through TRA1. Moreover, this correlation between the two genes decreases in tumor tissue, which may have implications on immune regulation mechanism, contributing to cancer progression. This shift could facilitate tumor evasion by promoting T-cell exhaustion or recruiting dysfunctional CD8 T-cell, which may explain our finding that high TRIM59 is significantly correlated with CD8+ T-cell infiltration in LIHC and KIRP, leading to poor prognosis.

In summary, protein interaction is a vital area with great potential for tumor diagnosis and therapy. However, further studies are needed to extend our understanding and maximize its benefits in these scopes.

Limitations

It is important to acknowledge the limitations of this study. To fully understand the implications of our findings, a larger number of datasets along with wet laboratory experiments are needed to examine the influence of TRIM59 and the potential mechanisms involved in carcinogenesis and prognosis across all cancer kinds, especially BRCA, ESCA, STAD, KIRP, LUSC, LIHC, and LUAD in order to validate our findings at the clinical level.

## Conclusions

The present study revealed that TRIM59 is upregulated in 22 tumors. BRCA, ESCA, stomach carcinoma, and LUSC expressed TRIM59 at significantly higher levels than normal tissues, making it a viable diagnostic biomarker for these cancers. Additionally, TRIM59 may serve as a potential prognostic marker for KIRP, LIHC, and LUAD. Moreover, our study is among the first studies that explored the role of TRIM59 expression on immune cell infiltration in BRCA, ESCA, LUSC, STAD, KIRP, LIHC, and LUAD. Also, this study suggested that the correlation between TRIM59 and P53 in BRCA, ESCA, STAD, LUSC, LUAD, and KIRP may contribute to carcinogenesis and increase the malignant behavior of these cancers. On the other hand, the correlation between TRIM59 and ABHD5 in BRCA, STAD, and LUAD as well as the correlation between TRIM59 and ECSIT in BRCA, ESCA, and KIRP are important and may be a potential area of therapeutic intervention. Collectively, TRIM59 can serve as a novel biomarker for the diagnosis of BRCA, ESCA, LUSC, and STAD as well as a prognostic biomarker for KIRP, LIHC, and LUAD. Furthermore, it holds promise as a potential therapeutic target for these cancers.
